# Host Cell Invasion and Virulence Mediated by *Candida albicans* Ssa1

**DOI:** 10.1371/journal.ppat.1001181

**Published:** 2010-11-11

**Authors:** Jianing N. Sun, Norma V. Solis, Quynh T. Phan, Jashanjot S. Bajwa, Helena Kashleva, Angela Thompson, Yaoping Liu, Anna Dongari-Bagtzoglou, Mira Edgerton, Scott G. Filler

**Affiliations:** 1 Department of Oral Biology, School of Dental Medicine, State University of New York at Buffalo, Buffalo, New York, United States of America; 2 Los Angeles Biomedical Research Institute at Harbor-UCLA Medical Center, Torrance, California, United States of America; 3 Division of Periodontology, School of Dental Medicine, University of Connecticut, Farmington, Connecticut, United States of America; 4 The David Geffen School of Medicine at UCLA, Los Angeles, California, United States of America; University of Massachusetts Medical School, United States of America

## Abstract

*Candida albicans* Ssa1 and Ssa2 are members of the HSP70 family of heat shock proteins that are expressed on the cell surface and function as receptors for antimicrobial peptides such as histatins. We investigated the role of Ssa1 and Ssa2 in mediating pathogenic host cell interactions and virulence. A *C. albicans ssa1*Δ/Δ mutant had attenuated virulence in murine models of disseminated and oropharyngeal candidiasis, whereas an *ssa2*Δ/Δ mutant did not. *In vitro* studies revealed that the *ssa1*Δ/Δ mutant caused markedly less damage to endothelial cells and oral epithelial cell lines. Also, the *ssa1*Δ/Δ mutant had defective binding to endothelial cell N-cadherin and epithelial cell E-cadherin, receptors that mediate host cell endocytosis of *C. albicans*. As a result, this mutant had impaired capacity to induce its own endocytosis by endothelial cells and oral epithelial cells. Latex beads coated with recombinant Ssa1 were avidly endocytosed by both endothelial cells and oral epithelial cells, demonstrating that Ssa1 is sufficient to induce host cell endocytosis. These results indicate that Ssa1 is a novel invasin that binds to host cell cadherins, induces host cell endocytosis, and is critical for *C. albicans* to cause maximal damage to host cells and induce disseminated and oropharyngeal disease.

## Author Summary

The fungus *Candida albicans* can proliferate in the mouth, causing oropharyngeal candidiasis. In other patients, it can enter the bloodstream and spread throughout the body, resulting in hematogenously disseminated candidiasis. Fungal invasion of host cells is a key feature of both types of infection. One mechanism by which *C. albicans* invades both the epithelial cell lining of the oropharynx and the endothelial cell lining of the blood vessels is by inducing its own uptake. This uptake is induced in part by the binding of the *C. albicans* invasin Als3 to host cell proteins, which include N- and E-cadherin. Here we show that *C. albicans* Ssa1, a member of the 70 kDa heat shock protein family, is expressed on the surface of *C. albicans* where it functions as an invasin. The key role of Ssa1 in host cell invasion is illustrated by the reduced capacity of an ssa1Δ/Δ null mutant to induce its own uptake by epithelial and endothelial cells in vitro, and by the significantly attenuated virulence of this mutant in mouse models of oropharyngeal candidiasis and disseminated candidiasis. Thus, Ssa1 is the second identified invasin of *C. albicans.*

## Introduction

The fungus, *Candida albicans* is a significant human pathogen. In hospitalized patients, this organism disseminates hematogenously and infects virtually all organs. Even with currently available therapy, bloodstream infections with *C. albicans* are associated with a 37% mortality [1]. *C. albicans* is also part of the normal oral flora and it usually grows as a harmless commensal. However, when local or systemic host defense mechanisms are impaired, this organism can proliferate and cause debilitating oropharyngeal candidiasis.

To persist within the human host and cause disease, *C. albicans* must be able to adhere to and invade host cells or tissues while resisting the stress caused by host-derived reactive oxygen intermediates and antimicrobial peptides [2]–[5]. In other organisms, heat shock proteins play an important role in each of these activities. For example, some heat shock proteins are expressed on the cell surface of microorganisms, where they function as adhesins [6]-[9]. Also, in some bacteria and parasites, members of the Hsp70 and Hsp100 family of heat shock proteins are required for resistance to host-induced stress [10]–[12].

Ssa1 and Ssa2 are the only two members of the Hsp70 family in *C. albicans,* and both proteins are expressed on the cell surface of yeast and hyphae [13], [14]. Previously, we found that histatin 5, one of the main antimicrobial proteins found in saliva, binds with high affinity to Ssa2 and with lower affinity to Ssa1. After histatin 5 is bound to Ssa proteins, it is transported into the cytoplasm, where it kills the fungal cell [15], [16]. *C. albicans* Ssa1 and Ssa2 are also required for maximal fungicidal activity of human β defensins 2 and 3 [17].

As reported here, we investigated the roles of Ssa1 and Ssa2 in *C. albicans* virulence in murine models of hematogenously disseminated and oropharyngeal candidiasis. We found that Ssa1, but not Ssa2 is essential for normal virulence in both models. Through *in vitro* experiments, we discovered that surface-expressed Ssa1 likely contributes to virulence by acting as an invasin and directly mediating *C. albicans* invasion of both endothelial and oral epithelial cells *in vitro*.

## Results

### Ssa1 is required for maximal virulence in a mouse model of hematogenously disseminated candidiasis

**Figure 1:**
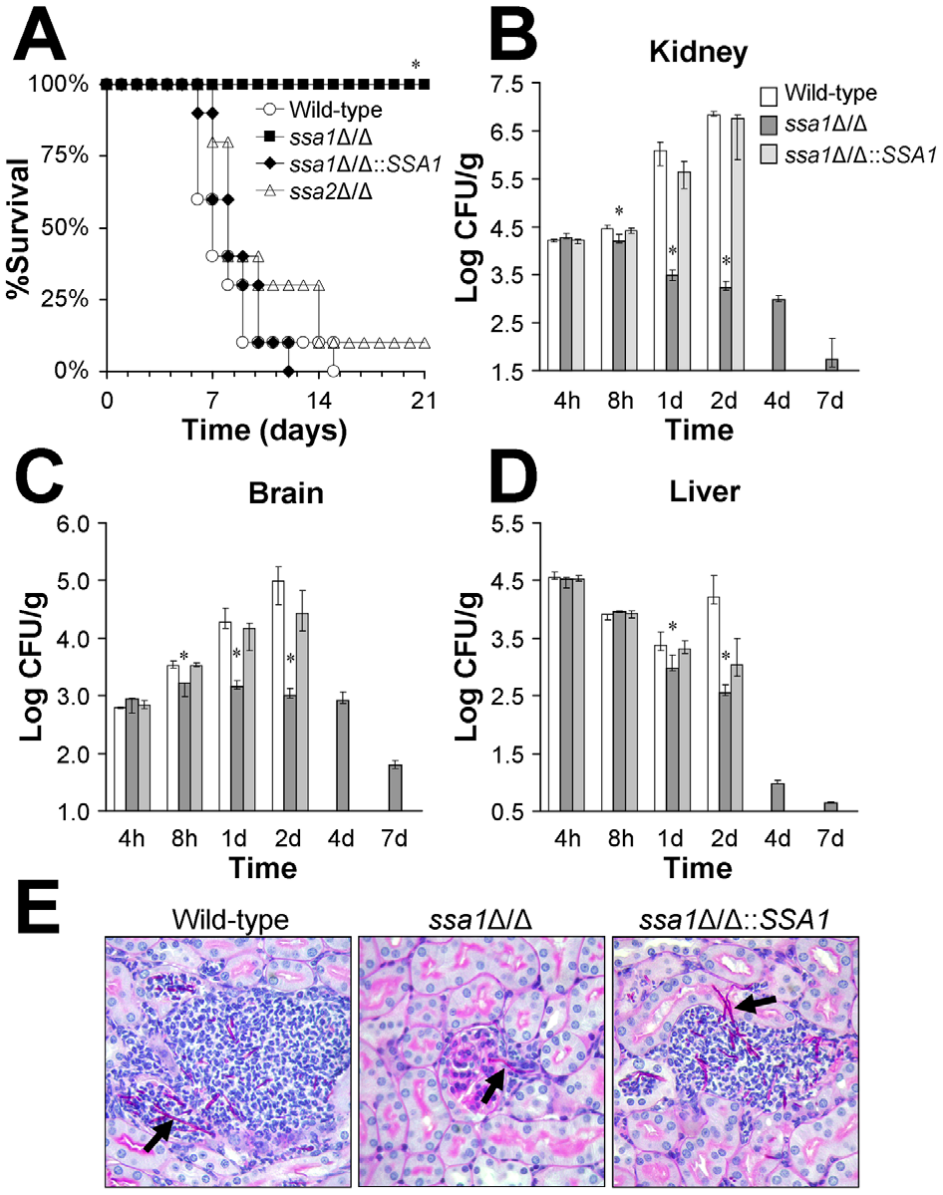
Ssa1 is required for normal virulence during hematogenously disseminated candidiasis. Mice were inoculated via the tail vein with 5×10^5^ yeast-phase cells of the indicated strains of *C. albicans*. (A) Survival over 21 days (n = 10 mice per strain of *C. albicans*). *P<0.0001 compared to mice infected with either the wild-type or *ssa1*Δ/Δ::*SSA1* complemented strain. (B–D) Fungal burden of the kidneys (B), brains (C), and livers (D) of mice infected with the wild-type, *ssa1*Δ/Δ and *ssa1*Δ/Δ::*SSA1* complemented strains at the indicated time points. For each organ, the lower bound of the y-axis scale indicates the limits of detection. Results from the 1 day time point are the median ± interquartile range of two experiments with a total of 12 mice per strain of *C. albicans*. Results from the other time points are the median ± interquartile range of one experiment with 7 mice per strain *P<0.02 compared to mice infected with either the wild-type or *ssa1*Δ/Δ::*SSA1* complemented strain. (E) Periodic acid Schiff stained thin sections of kidneys after 1 day of infection with the indicated strains of *C. albicans*. Arrows indicate *C. albicans* filaments in the tissues.

To evaluate the contribution of Ssa1 and Ssa2 to *C. albicans* pathogenicity, the virulence of *ssa1*Δ/Δ and *ssa2*Δ/Δ null mutants was tested in a murine model of disseminated candidiasis. Over the 21 day observation period, none of the mice infected intravenously with the *ssa1*Δ/Δ mutant died (Figure 1A). In contrast, the median survival of mice infected with the wild-type and *ssa1*Δ/Δ::*SSA1*Δ/Δ complemented strain was 7 to 8 days. Ssa2 did not appear to influence virulence in this model because the survival of mice infected with the *ssa2*Δ/Δ null mutant was similar to that of mice infected with the wild-type strain (*p = *0.13).

Next, we compared the organ fungal burden at various time points in mice infected with the different strains. After 8 h of infection, the kidney and brain fungal burdens of mice inoculated with the *ssa1*Δ/Δ mutant were significantly lower than mice infected with either the wild-type or *ssa1*Δ/Δ::*SSA1*Δ/Δ complemented strains (Figures 1B–D). After 1 and 2 days of infection, the kidney and brain fungal burdens of mice challenged with the *ssa1*Δ/Δ mutant either declined or were stable. In contrast, the fungal burden of these organs steadily increased in mice infected with either the wild-type or *ssa1*Δ/Δ::*SSA1*Δ/Δ complemented strains. The fungal burdens of the livers of mice infected with the *ssa1*Δ/Δ mutant were also significantly lower than those of the control mice after 1 and 2 days of infection. These results indicate that *SSA1* is required for normal levels of *C. albicans* infection in the kidney and brain as early as 8 h after inoculation. *SSA1* also appears to be necessary for the organism to persist in the tissues at later time points.

To investigate why mice infected with the *ssa1*Δ/Δ mutant had no mortality over the course of the infection, we determined their organ fungal burden after 4, 7 and 14 days post-inoculation. We were not able to analyze the organ fungal burden of mice infected with either the wild-type strain or the *ssa1*Δ/Δ::*SSA1*Δ/Δ complemented strain at these later time points because a significant percentage of these mice had died. We found that mice infected with the *ssa1*Δ/Δ mutant progressively cleared the infection so that their organs became sterile by 14 days (Figure 1B–D and data not shown). This clearance provides an explanation for the prolonged survival of mice infected with the *ssa1*Δ/Δ mutant.

The low fungal burden in the kidneys of mice infected with the *ssa1*Δ/Δ mutant was verified by histopathology. After 1 day of infection, the kidneys of these mice contained very small foci of organisms. These foci usually consisted of a single fungal element surrounded by a few inflammatory cells (Figure 1E). Interestingly, many of the *ssa1*Δ/Δ cells were located in the glomeruli. The kidneys from mice infected with either the wild-type or *ssa1*Δ/Δ::*SSA1*Δ/Δ complemented strain contained the expected microabscesses, which contained numerous neutrophils surrounding multiple *C. albicans* filaments. It was not possible to determine whether the filaments of the various *C. albicans* strains were true hyphae or pseudohyphae in these histopathologic specimens. However, the filaments of the *ssa1*Δ/Δ mutant appeared to be similar in length to those of the wild-type and *ssa1*Δ/Δ::*SSA1*Δ/Δ complemented strain. Also, we have previously found that the *ssa1*Δ/Δ mutant forms hyphae similar to the wild-type strain in the presence of serum in vitro [16]. Therefore, the attenuated virulence of the *ssa1*Δ/Δ mutant was unlikely due to a defect in filamentation.

During the construction of the *ssa1*Δ/Δ mutant, *URA3* was used as the selectable marker. The chromosomal locus at which *URA3* is integrated can sometimes influence the virulence of *C. albicans* mutants in the mouse model of disseminated candidiasis [18]–[20]. In the *ssa1*Δ/Δ mutant, *URA3* was integrated at the *SSA2* locus [16]. However, in the *ssa1*Δ/Δ::*SSA1* complemented strain, *URA3* was integrated at the *RPS10* locus, which is known to result in normal activity of the *URA3* gene product, orotidine 5′-monophosphate decarboxylase [21]. To verify that the observed attenuated virulence of the *ssa1*Δ/Δ mutant was due to the absence of *SSA1* and not the result of the chromosomal locus of *URA3*, we tested the virulence of a second *ssa1*Δ/Δ strain in which *URA3* was integrated at the *RPS10* locus. As expected, all mice infected intravenously with this mutant survived (Supplemental Figure S1A), thus confirming that *SSA1* is required for the normal virulence of *C. albicans.*

### The *ssa1*Δ*/*Δ mutant had attenuated virulence in the mouse model of oropharyngeal candidiasis

**Figure 2:**
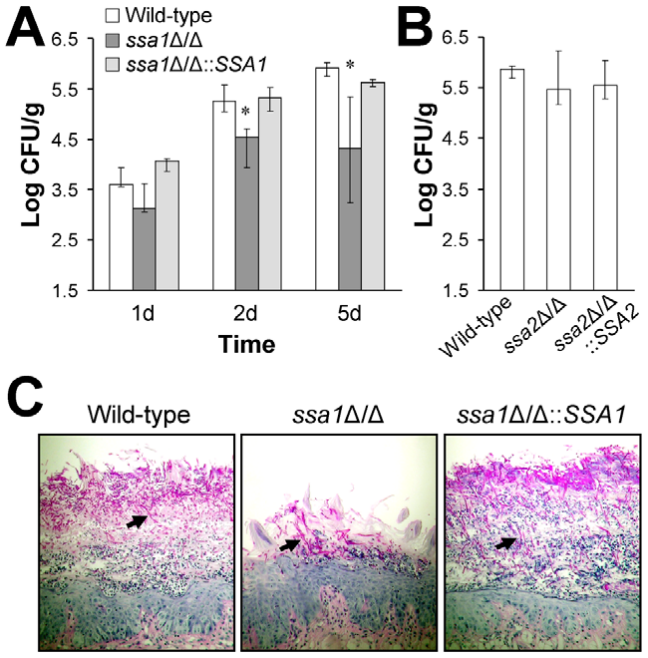
Attenuated virulence of the *ssa1*Δ/Δ mutant during oropharyngeal candidiasis. Mice were immunosuppressed with cortisone acetate and then orally inoculated with yeast-phase cells of the indicated strains of *C. albicans*. After 1, 2, and 5 days of infection, the mice were sacrificed and the tongues and attached tissues were excised. (A) Oral fungal burden of mice infected with the wild-type, *ssa1*Δ/Δ, and *ssa1*Δ/Δ::*SSA1* complemented strains for the indicated times. Results from the 1 and 2 day time points are the median ± the interquartile range of one experiment with 7 mice per strain of *C. albicans*. Results from the 5 day time point are the median ± the interquartile range of two experiments with a total of 12 mice per strain. *P≤0.01 compared to both the wild-type and *ssa1Δ/Δ::SSA1* strains. (B) Oral fungal burden of mice infected with the wild-type, *ssa2*, and *ssa2*Δ/Δ::*SSA2* complemented strain for 5 days. Results are the median ± the interquartile range of one experiment with 7 mice per strain of *C. albicans*. (C) Periodic acid-Schiff stained thin sections of the tongues of mice infected with the indicated strains for 5 days. Arrows indicate the *C. albicans* filaments.

Next, we investigated the contributions of *SSA1* and *SSA2* to virulence in a mouse model of oropharyngeal candidiasis. There was a trend towards reduced oral fungal burden in mice infected with the *ssa1*Δ/Δ mutant after 1 day of infection, but this difference was only significant when compared to the *ssa1*Δ/Δ complemented strain (*p* = 0.04), but not the wild-type strain (*p* = 0.13) (Figure 2A). However, after 2 and 5 days of infection, mice infected with the *ssa1*Δ/Δ mutant had markedly lower oral fungal burdens compared to mice infected with either the wild-type or *ssa1Δ/Δ::SSA1* complemented strain. In contrast, the oral fungal burden of mice infected with the *ssa2*Δ/Δ mutant was similar to that of mice infected with the wild-type strain (*p* = 0.57) (Figure 2B). Therefore, Ssa1 is required for maximal virulence during oropharyngeal candidiasis, but Ssa2 is dispensable for virulence during this infection.

The results of histopathologic examination of the tongues of the mice infected with the various strains verified the reduced virulence of the *ssa1*Δ/Δ mutant. The tongues of mice infected with the *ssa1*Δ/Δ mutant had relatively small lesions that contained fewer fungal cells and neutrophils than the lesions of mice infected with either the wild-type or the *ssa1Δ/Δ::SSA1* complemented strain (Figure 2C). Importantly, the filaments of the *ssa1*Δ/Δ mutant were similar in length to those of the wild-type strain. Collectively, these results indicate that Ssa1 is necessary for maximal *C. albicans* virulence during both hematogenously disseminated and oropharyngeal candidiasis.

### Ssa1 is required for *C. albicans* to cause maximal damage to both endothelial and oral epithelial cells in vitro

**Figure 3:**
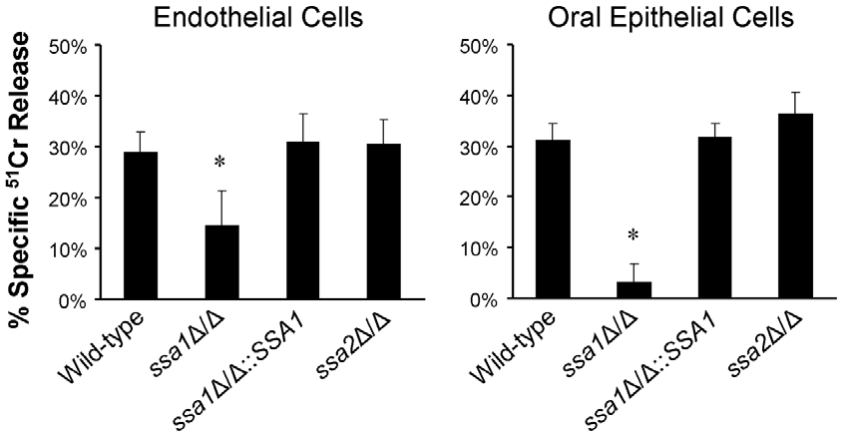
Ssa1 is necessary for *C. albicans* to cause maximal damage to endothelial cells and an oral epithelial cell line. Endothelial cells and the FaDu oral epithelial cell line were incubated with the indicated strains of *C. albicans* for 3 h, after which the extent of host cell damage was determined using a ^51^Cr release assay. Results are the mean ± SD of three independent experiments, each performed in triplicate. *P<0.001 compared with the wild-type and *ssa1*Δ/Δ::*SSA1* complemented strains.

During hematogenously disseminated candidiasis, blood-borne *C. albicans* cells must adhere to and penetrate the endothelial cell lining of the blood vessels to invade the deep tissues [3]. *C. albicans* also adheres to and invades oral epithelial cells during oropharyngeal candidiasis [22]–[24]. After the organism invades endothelial or oral epithelial cells *in vitro*, it damages these cells. Moreover, *C. albicans* mutants with impaired capacity to cause host cell damage *in vitro* frequently have attenuated virulence in mice [25]–[27]. Therefore, we used a ^51^Cr release assay to determine the capacity of the *ssa1*Δ/Δ mutant to damage monolayers of human umbilical vein endothelial cells and the FaDu oral epithelial cell line. In this assay, the host cells are incubated with ^51^Cr, which is taken up by the cells and binds to cytoplasmic proteins. When the host cells are damaged, there is loss of membrane integrity and the labeled proteins leak out of the cells into the medium. The amount of ^51^Cr that is released is proportional to the extent of cellular damage [26]–[31]. We found that the *ssa1Δ/Δ* mutant caused 50% less damage to endothelial cells and 89% less damage to epithelial cells compared to the wild-type strain (Figure 3). Complementing the *ssa1Δ/Δ* mutant with a wild-type copy of *SSA1* restored its capacity to damage these cells. As predicted by the virulence data, the *ssa2*Δ/Δ mutant caused the same extent of damage to the endothelial and epithelial cells as did the wild-type strain. All strains of *C. albicans* formed true hyphae that were of similar length on both endothelial and epithelial cells (data not shown). Therefore, the host cell damage defects of the *ssa1*Δ/Δ mutant were not due to impaired hyphal formation [27], [32], [33]. These results indicate that Ssa1 is necessary for *C. albicans* to cause maximal damage to endothelial and oral epithelial cells *in vitro*. This defective capacity to damage host cells likely contributed to the attenuated virulence of the *ssa1*Δ/Δ mutant.

**Figure 4:**
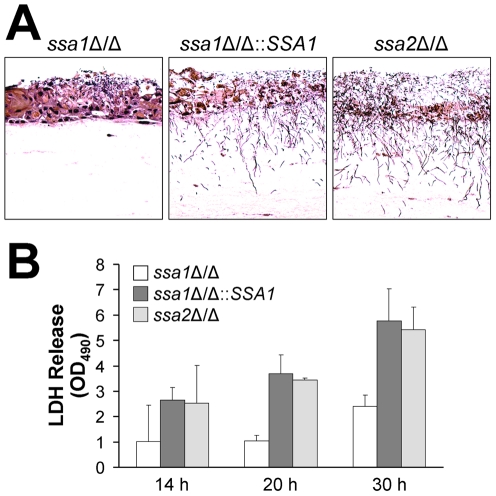
The *ssa1*Δ/Δ mutant has impaired capacity to damage a three-dimensional organotypic model of the oral mucosa. The three-dimensional model of the oral mucosa was infected by adding 10^5^ yeast-phase cells of the indicated *C. albicans* strains to the apical surface. (A) Histopathology after 2 days of infection. Thin sections were stained with periodic acid-Schiff. The results are representative of one of three experiments. (B) Epithelial cell damage by the indicated strains was quantified by the release of lactate dehydrogenase (LDH) into the medium. Results are the mean ± SD of 2 experiments.

Although endothelial cells grow as a monolayer *in vivo*, epithelial cells grow in multiple, stratified layers in the oropharynx. Therefore, we tested the interactions of the *ssa1*Δ/Δ and *ssa2*Δ/Δ mutants with oral epithelial cells in a three-dimensional culture model, which more closely mimics the normal human oral mucosa and submucosa [34]. Consistent with the intraoral animal findings, the *ssa1Δ/Δ* mutant grew only in small foci and caused little visible epithelial cell damage, even after 2 days of infection (Figure 4A). There was only slight cellular edema in the epithelium, which otherwise maintained its barrier function and prevented the organisms from crossing the basal layer of cells into the collagen gel. In contrast, the *ssa1*Δ/Δ::*SSA1* complemented strain formed a thick biofilm on the epithelial cells, which resulted in degradation of most of the epithelial layers and subsequent submucosal invasion. As predicted by our previous results, the *ssa2Δ/Δ* strain induced extensive epithelial destruction and invaded into the submucosal compartment similarly to the *ssa1*Δ/Δ::*SSA1* complemented strain.

To quantify the extent of epithelial cell damage caused by the various strains of *C. albicans* in the 3-dimensional model, we measured the leakage of lactate dehydrogenase (LDH) into the medium. The *ssa1*Δ/Δ mutant induced less LDH release than did the *ssa1*Δ/Δ::*SSA1* complemented or *ssa2Δ/Δ* strains (Figure 4B). These results provide further support for the importance of Ssa1 in the capacity of *C. albicans* to damage oral epithelial cells.

### Ssa1 governs the capacity of *C. albicans* to bind to cadherins and invade endothelial and oral epithelial cells

**Figure 5:**
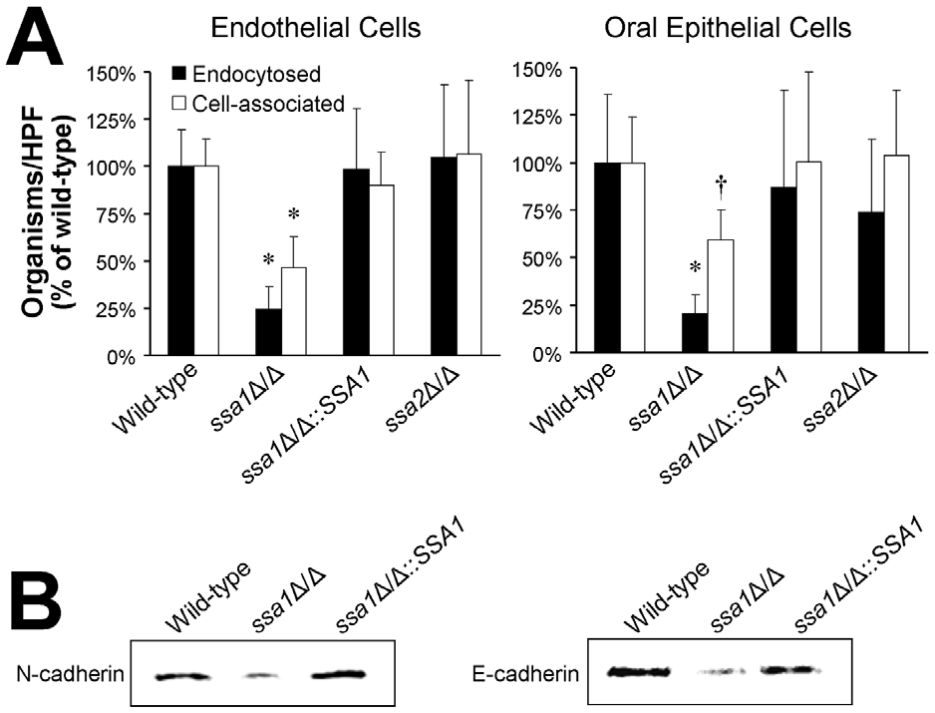
Ssa1 is necessary for *C. albicans* to adhere to and be endocytosed by endothelial cells and oral epithelial cells. (A) Adherence and endocytosis assay. The indicated strains of *C. albicans* were incubated with endothelial cells or FaDu oral epithelial cells for 90 min, after which the number of endocytosed and cell-associated (endocytosed plus adherent) organisms was determined by a differential fluorescent assay. Results are the mean ± SD of three independent experiments, each performed in triplicate. *P<0.005 compared to the wild-type and *ssa1*ΔΔ::*SSA1* complemented strains; ^†^ *p*<0.03 compared to the wild-type and *ssa1*ΔΔ::*SSA1* complemented strains. (B) The capacity of the indicated strains to bind to N-cadherin in extracts of endothelial cell membrane proteins and E-cadherin in extracts of FaDu oral epithelial cell membrane proteins was determined using a whole-cell affinity purification approach. The immunoblot of endothelial cell proteins eluted from hyphae of the indicated strains was developed with an anti-N-cadherin antibody. The immunoblot of epithelial cell proteins eluted from hyphae of the indicated strains was developed with an anti-E-cadherin antibody.

To cause maximal damage endothelial or oral epithelial cells, *C. albicans* must adhere to and then invade these host cells [27], [30], [31]. One mechanism by which *C. albicans* invades endothelial cells and oral epithelial cells is by inducing its own endocytosis [27], [31], [33], [35]. Therefore, we used our standard differential fluorescent assay to determine if the *ssa1*Δ/Δ mutant had a defect in its capacity to adhere to and/or be endocytosed by endothelial and FaDu epithelial cells *in vitro*. Compared to the wild-type strain, the *ssa1Δ/Δ* mutant was endocytosed poorly by both endothelial cells and oral epithelial cells (Figure 5A). The *ssa1*Δ/Δ::*SSA1* complemented strain and the *ssa2*Δ/Δ mutant interacted with both the endothelial and epithelial cells similarly to the wild-type strain. In addition, endocytosis defects of the *ssa1*Δ/Δ mutant persisted when *URA3* was integrated at the *RPS10* locus (Supplemental Figure S1B). Therefore, Ssa1, but not Ssa2, is required for *C. albicans* to invade endothelial cells and oral epithelial cells *in vitro*.

*C. albicans* induces its own endocytosis by endothelial cells *in vitro* by binding to N-cadherin on the endothelial cell surface [36]. The corresponding receptor for *C. albicans* on oral epithelial cells is E-cadherin [37]. To determine the mechanism of host cell invasion defects of the *ssa1*Δ/Δ mutant, we examined its capacity to bind to N-cadherin and E-cadherin in cell membrane extracts from endothelial cells and epithelial cells, respectively. Hyphae of the *ssa1*Δ/Δ mutant bound poorly to N-cadherin and E-cadherin relative to both the wild-type and *ssa1*Δ/Δ::*SSA1* complemented strain, (Figure 5B). This reduced binding to cadherins likely contributed to the impaired host cell invasion and subsequent reduced host cell damage of the *ssa1*Δ/Δ mutant.

### Ssa1 can directly mediate adherence to and endocytosis by host cells

**Figure 6:**
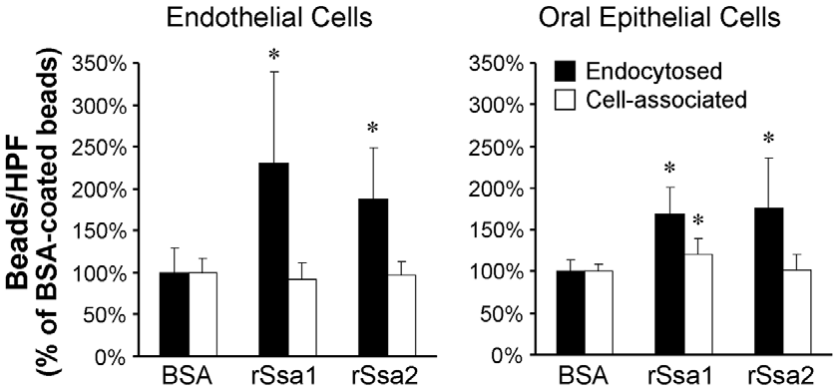
Ssa1 is sufficient to induce endocytosis by host cells. Latex beads were coated with bovine serum albumin (BSA), recombinant Ssa1 (rSsa1), or rSsa2. They were incubated for 45 min with endothelial cells and FaDu oral epithelial cells, after which the number of endocytosed and cell-associate beads was determined. Data are expressed as a percentage of the results with beads coated with BSA and are the mean ± SD of 3 experiments, each performed in triplicate. *P<0.01 compared to beads coated with BSA.

Ssa1 is expressed either on or near the cell surface of both hyphae and yeast phase *C. albicans* [13]. Therefore, we investigated the possibility that Ssa1 by itself could mediate pathogenic host cell interactions. Latex beads were coated with recombinant Ssa1 (rSsa1), rSsa2, or bovine serum albumin (BSA), after which their endocytosis by host cells was measured. Both endothelial cells and epithelial cells endocytosed significantly more beads coated with either rSsa1 or rSsa2 compared to the control BSA-coated beads (Figure 6). These results suggest that both Ssa1 and Ssa2 can directly induce endocytosis by endothelial and epithelial cells.

**Figure 7:**
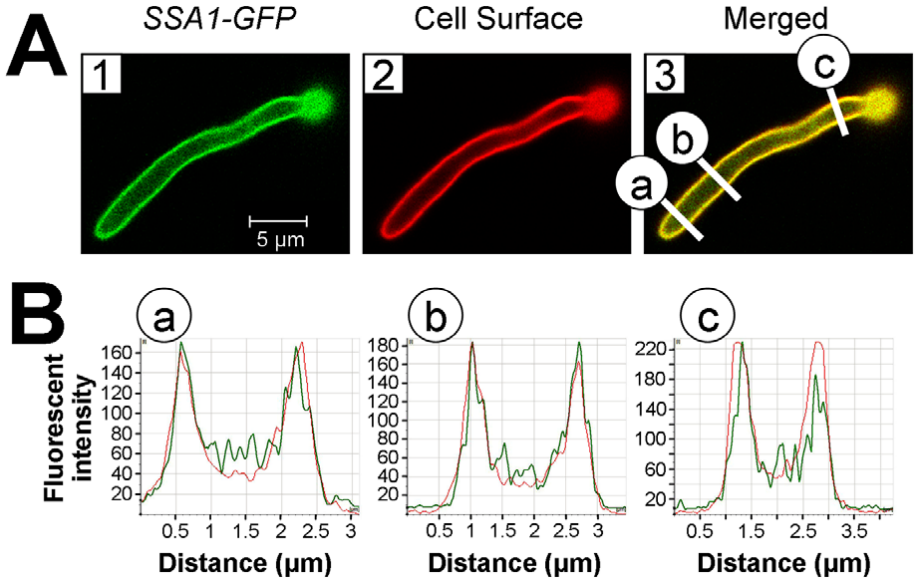
Ssa1 is located on the cell surface of *C. albicans* hyphae during epithelial cell interaction. FaDu oral epithelial cells were infected with *C. albicans* expressing an Ssa1-GFP fusion protein for 90 min, after which the cells were fixed and stained with an Alexa 594-conjugated anti-*C. albicans* polyclonal antibody to label the cell surface. (A) Confocal microscopic images of Ssa1-GFP (1) and fluorescent-labeled anti-*C. albicans* antibody (2). The merged image is shown in (3) and the regions in yellow indicate areas of co-localization between Ssa1-GFP and the fluorescent-labeled anti-*C. albicans* antibody. (B) Graphs of fluorescent intensity at different cross sections of the hypha in panel (3). The green lines indicate the fluorescent intensity of the Ssa1-GFP and the red lines indicate the fluorescent intensity of the fluorescent-labeled anti-*C. albicans* antibody. The letters (a-c) correspond to those in panel (3) and indicate the locations along the hypha at which the fluorescent intensity was measured.

To verify that Ssa1 was expressed on the surface of *C. albicans* hyphae that were interacting with host cells, we infected FaDu epithelial cells with a strain of *C. albicans* that expressed an Ssa1-GFP fusion protein. After a 90 min incubation, the cells were fixed, stained with an Alexa 594-conjugated anti-*C. albicans* antibody to label the cell surface, and then imaged by confocal microscopy. We found that Ssa1-GFP fluorescence was strongest at the periphery of the hyphae (Figure 7). Importantly, this fluorescence co-localized with that of the fluorescently-labeled anti-*C. albicans* antibody, indicating that Ssa1 was located in the outermost part of the cell wall. Interestingly, when a similar experiment was performed using organisms grown as yeast cells in suspension, Ssa1 was located slightly internal to the cell surface (Figure S2). These results suggest that the location of Ssa1 is dependent on the morphology and culture conditions of the organism. Importantly, when *C. albicans* hyphae are in contact with host cells, Ssa1 is likely expressed on the cell surface where it can interact with host cell receptors.

### Ssa1 and Als3 function in the same pathway(s) to induce endocytosis

**Figure 8:**
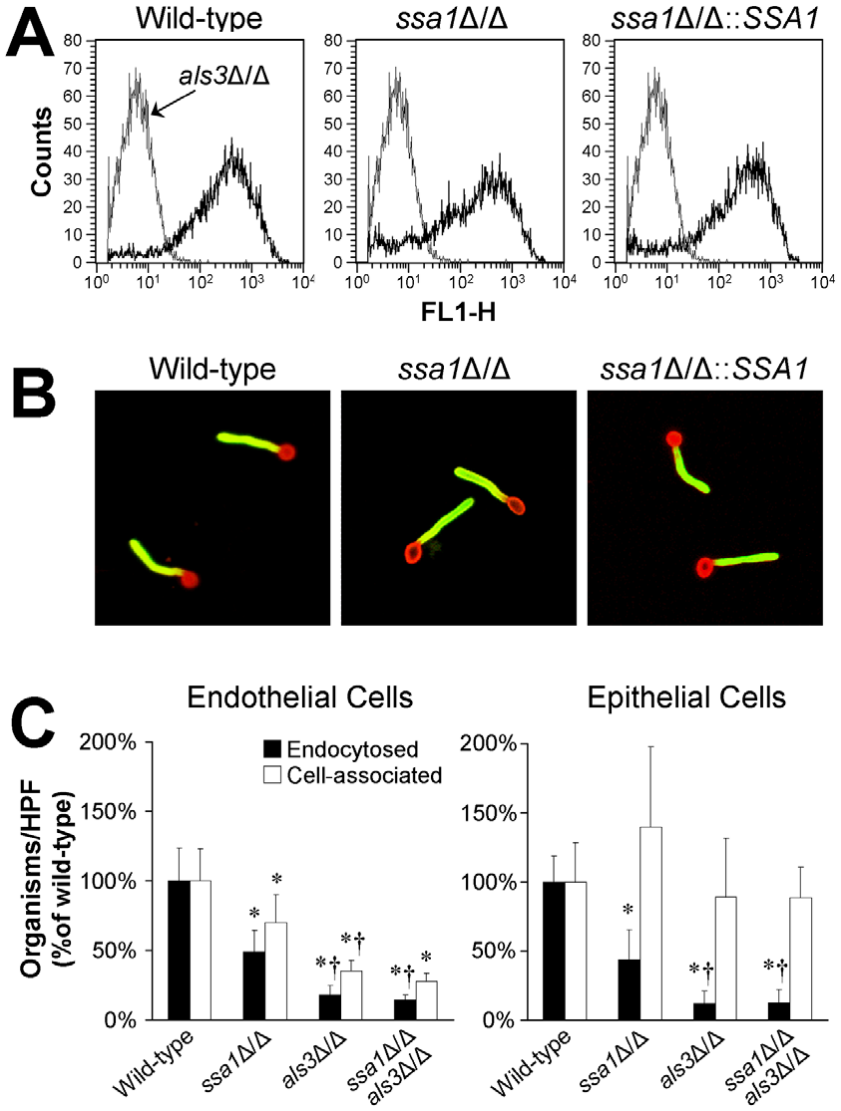
Interaction between Ssa1 and Als3. (A) The *ssa1*Δ/Δ mutant has normal amounts of Als3 on its surface. Hyphae of the indicated *C. albicans* strains were stained with a polyclonal anti-Als3 antibody, after which amount of surface exposed Als3 was determined by flow cytometric analysis of 10,000 cells per strain. (B) Normal surface distribution of Als3 on *ssa1*Δ/Δ hyphae. The distribution of Als3 on the surface of hyphae of the indicated *C. albicans* strains was determined by staining the organisms with a polyclonal anti-Als3 antibody (green fluorescence) and counterstaining with an anti-*C. albicans* polyclonal antibody (red fluorescence). (C) Effects of deletion of *ALS3* in the *ssa1*Δ/Δ mutant background. Endothelial cells and FaDu oral epithelial cells were incubated with the indicated strains for 150 min, after which the number of endocytosed and cell-associated organisms were determined. Results are the mean ± SD of 3 experiments, each performed in triplicate. *P<0.01 compared to the wild-type strain, ^†^ *p*<0.005 compared to the *ssa1*Δ/Δ single mutant.

One *C. albicans* ligand that binds to N-cadherin and E-cadherin is Als3, and *als3*Δ/Δ mutants have significantly reduced adherence to and invasion of both endothelial cells and oral epithelial cells in vitro [37]–[39]. Because heat shock proteins can potentially influence the expression of other proteins on the surface of *C. albicans*, we investigated the possibility that the host cell interaction defects of the *ssa1*Δ/Δ mutant were due to decreased surface expression of Als3. Flow cytometric analysis of hyphae stained with a polyclonal anti-Als3 antibody demonstrated that the *ssa1*Δ/Δ mutant expressed a similar amount of Als3 on its surface as did the wild-type and *ssa1*Δ/Δ::*SSA1* complemented strains (Figure 8A). Furthermore, using indirect immunofluorescence with the anti-Als3 antibody, we determined that Als3 was distributed along the entire length of *ssa1*Δ/Δ mutant hyphae, similar to the wild-type and *ssa1*Δ/Δ::*SSA1* complemented strains (Figure 8B). Thus, the host cell interaction defects of the *ssa1*Δ/Δ mutant are unlikely to be due to abnormal amount or location of Als3 on the cell surface.

To further investigate possible interactions between Ssa1 and Als3, we constructed an *ssa1*Δ/Δ *als3*Δ/Δ double mutant and compared its host cell interactions with those of *ssa1*Δ/Δ and *als3*Δ/Δ single mutants. When a 90 min incubation period was used, the host cell interaction defects of the both the *ssa1*Δ/Δ and *als3*Δ/Δ mutants were so large that it was not possible to detect any further reduction in endocytosis of the *ssa1*Δ/Δ *als3*Δ/Δ double mutant (Figure 5 and data not shown). Therefore, we increased the incubation period to 150 min. We found that at this time point, the *als3*Δ/Δ single mutant had a greater defect in inducing endocytosis by endothelial and epithelial cells than did the *ssa1*Δ/Δ single mutant (Figure 8C). However, the endocytosis defect of the *ssa1*Δ/Δ *als3*Δ/Δ double mutant was similar to that of the *als3*Δ/Δ single mutant. Collectively, these results indicate that Ssa1 and Als3 function in the same pathway(s) to induce endocytosis, perhaps by binding to the same host cell surface proteins and/or forming part of the same multiprotein complex.

### *SSA1* is expressed at higher levels than *SSA2*

**Figure 9:**
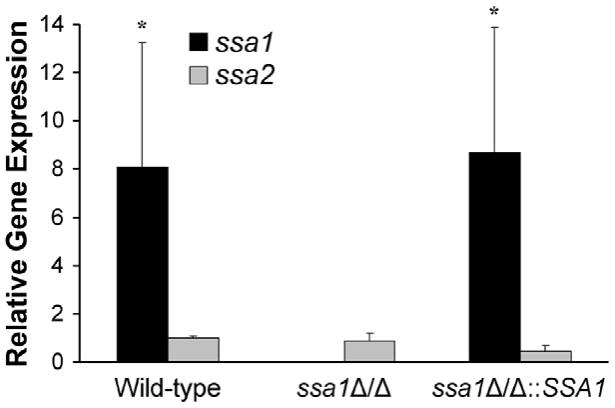
Relative transcript levels of *SSA1* and *SSA2*. FaDu epithelial cells were infected with the indicated strains of *C. albicans* for 90 min, after which the *C. albicans* RNA was extracted. The relative transcript levels of *SSA1* and *SSA2* were measured by real-time PCR. Results are the mean ± SD of three biological replicates, each tested in duplicate. *P<0.01 compared to *SSA2* transcript levels.

It seemed paradoxical that Ssa2 was sufficient to induce endocytosis by host cells, yet the *ssa2*Δ/Δ mutant was endocytosed similarly to the wild-type strain. One potential explanation for these results is that *SSA1* is expressed at a higher level than *SSA2*. Previously, we had found that, in yeast-phase *C. albicans*, the *SSA1* mRNA transcript levels were significantly greater than those of *SSA2*, and the fungal cell wall contained 4- to 5- fold more Ssa1 than Ssa2 [16]. To determine if *SSA1* was expressed greater than *SSA2* in hyphae that were in contact with host cells, we infected FaDu oral epithelial cells with the various *C. albicans* strains and allowed them to germinate. *SSA1* and *SSA2* mRNA expression in these organisms was measured by real-time PCR. We found that in the wild-type strain, *SSA1* was expressed 8-fold higher than *SSA2* (Figure 9). As expected, *SSA1* mRNA was not detectable in the *ssa1*Δ/Δ mutant. Importantly, *SSA2* expression in this mutant was similar to that of the wild-type strain, indicating that *SSA2* was not up-regulated in compensation for the absence of *SSA1*. Therefore, even though Ssa2 is capable of stimulating host cell endocytosis, the effects of *SSA2* deletion are likely masked by the high level expression of *SSA1.*

### Ssa1 is not required for secretion of phospholipases and proteases

**Figure 10:**
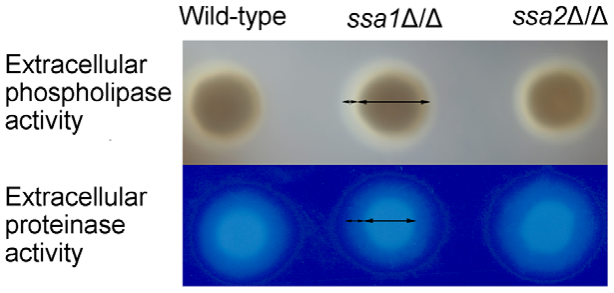
*C. albicans* extracellular phospholipase and protease activities are independent of Ssa1 and Ssa2. The total extracellular phospholipase activity of the indicated strains was determined by growing them on egg yolk agar at room temperature for 5 days and then measuring the width of the zone of precipitation around each colony (top panel). The total extracellular protease activity of the indicated strains was assessed by growing them on BSA agar at 37°C for 5 days and then staining the plates with 0.5% amido black. The extracellular protease activity was proportional to the width of the zone of clearance around the colonies (lower panel).

*C. albicans* secretes phospholipases and aspartyl proteases, both of which contribute to the virulence of this organism, probably by damaging host cells [35], [40]–[46]. Heat shock proteins can influence protein secretion [47], [48]. Therefore, we investigated the possibility that the attenuated damage of endothelial and epithelial cells caused by the *ssa1*Δ/Δ mutant was due to reduced secretion of phospholipases or proteases. We grew the various strain on either egg yolk agar or BSA agar and measured the size of the zones of precipitation or clearance around the colonies to screen for total extracellular phospholipase and protease activity, respectively [43], [49]. The *ssa1*Δ/Δ mutant was similar to the wild-type and *ssa1*Δ/Δ::*SSA1* complemented strains in these assays (Figure 10). These data suggest that the reduced virulence of the *ssa1*Δ/Δ mutant was unlikely the result of diminished secretion of phospholipases or proteases.

### *C. albicans* resistance to stress is independent of Ssa1 and Ssa2

**Figure 11:**
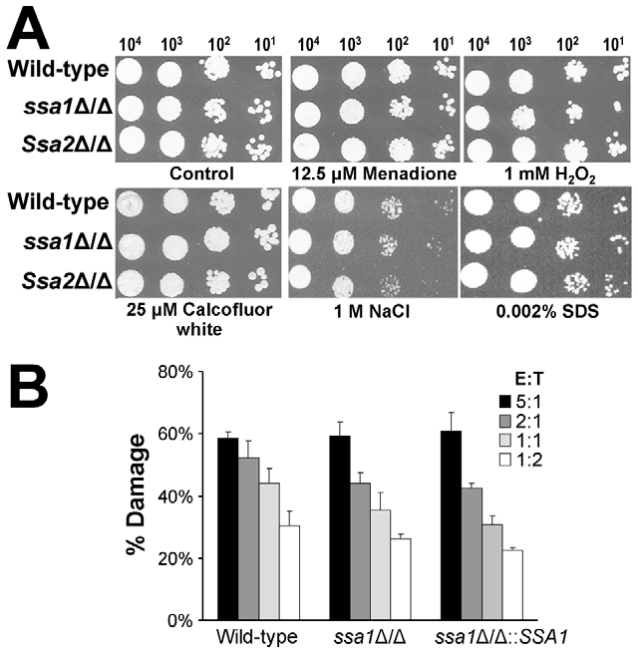
Ssa1 and Ssa2 are not required for resistance to stress. (A) Susceptibility to oxidant and cell wall stress. Serial 10-fold dilutions of the indicated strains were used to inoculate YPD plates containing menadione, H_2_O_2_, Calcofluor white, NaCl, or SDS. Images were obtained after growth at 30°C for 24 h. (B) Susceptibility to leukocyte induced damage. Yeast-phase cells of the indicated strains were incubated for 3 h with HL-60 cells that had been differentiated into neutrophil-like cells, after which the amount of damage to the organisms was determined by an XTT assay. Each strain was tested at an effector to target ratio (E:T) ranging from 5∶1 to 1∶2. Results are the mean ± SD of three independent experiments, each performed in triplicate.

Heat shock proteins are important for some microorganisms to resist the stressful conditions they encounter in the host [10]–[12]. Therefore, increased susceptibility to host-induced stress could have contributed to the reduced virulence of the *ssa1*Δ/Δ mutant. To evaluate this possibility, we tested the susceptibility of the *ssa1*Δ/Δ and *ssa2*Δ/Δ mutants to various stressors. Both the *ssa1*Δ/Δ and *ssa2*Δ/Δ mutants had wild-type susceptibility to oxidant (menadione and H_2_O_2_), cell wall (Calcofluor white), osmotic (NaCl), and plasma membrane (SDS) stress (Figure 11A). To verify that deletion of *SSA1* or *SSA2* did not affect stress resistance, we tested these stressors at higher concentrations. The growth of the *ssa1*Δ/Δ and *ssa2*Δ/Δ mutants was inhibited similarly to the wild-type strain under these conditions (data not shown). In addition, all strains had comparable susceptibility to nitric oxide (data not shown). We also tested the susceptibility of the *ssa1*Δ/Δ mutant to damage by the human neutrophil-like HL-60 cell line. This mutant was not more susceptible than the wild-type and *ssa1*Δ/Δ::*SSA1* complemented strains (*p*≥0.12) (Figure 11B). Therefore, the reduced virulence of the *ssa1*Δ/Δ mutant was unlikely to be due to increased susceptibility to host induced stress.

## Discussion

Heat shock proteins play diverse roles in the pathogenicity of many microorganisms, including bacteria, protozoa, and fungi [6]–[12]. However, the contribution of members of the HSP70 family of heat shock proteins to *C. albicans* virulence has not been reported previously. Our results with the *ssa1*Δ/Δ mutant demonstrate that Ssa1 is require for maximal *C. albicans* virulence during both hematogenously disseminated and oropharyngeal candidiasis in mice.

The data from our *in vitro* studies indicated that the *ssa1*Δ/Δ mutant was defective in its capacity to adhere to, invade, and damage both endothelial cells and oral epithelial cells. The invasion defect of this mutant was likely due in part to its impaired capacity to bind to endothelial cell N-cadherin and epithelial cell E-cadherin, receptors that can mediate the endocytosis of *C. albicans* [36], [37], [39]. Other *C. albicans* mutants that are defective in invading and damaging endothelial cells or oral epithelial cells frequently have attenuated virulence in mouse models of candidiasis [25], [26], [32], [50]. Therefore, it is highly probable that the host cell interaction defects of the *ssa1*Δ/Δ mutant contributed to its attenuated virulence in mice.

The experiments with latex beads coated with Ssa1 or Ssa2 demonstrated that either protein can induce endocytosis by both endothelial cells and oral epithelial cells. However, in intact organisms, Ssa1 is likely more important than Ssa2 for inducing host cell endocytosis because the transcript levels of *SSA1* are much greater. In addition, using GFP-labeled Ssa1, we verified that Ssa1 is expressed on the surface of hyphae that are in contact with epithelial cells. Thus, Ssa1 is located where it can bind to host cell receptors. Collectively, these results indicate that Ssa1 can function as an invasin and induce host cells to endocytose the organism. Members of the Hsp70 family have also been found to mediate the host cell adherence of bacteria such as *Helicobacter pylori*, *Haemophilus influenza*, and *Mycobacterium avium* [6]–[8]. In addition, Hsp60 is expressed on the surface of *Histoplasma capsulatum* and is the major ligand for CD11b/CD18 on macrophages [9]. Orthologs of Ssa1 and Ssa2 are also present in species of *Candida* other than *C. albicans*. Whether these orthologs also mediate pathogenic host cell interactions remains to be determined.

Ssa1 is functionally similar to the *C. albicans* invasin Als3, which also binds to cadherins on the surface of endothelial cells and epithelial cells, and thereby induces endocytosis [37], [39]. It is notable that *ssa1*Δ/Δ and *als3*Δ/Δ mutants both have defects in their capacity to invade and damage these cells *in vitro.* Furthermore, the host cell invasion defect of the *ssa1*Δ/Δ *als3*Δ/Δ double mutant was similar that of the *als3*Δ/Δ single mutant. The most likely explanation for this result is that Ssa1 and Als3 bind to same endothelial and epithelial cell surface proteins, either separately or perhaps as part of multiprotein complex. An alternative explanation for these results is that Ssa1 functions as a molecular chaperone that is required for appropriate folding or trafficking of Als3 [47], [48], [51], [52]. Although we cannot completely exclude the possibility that the folding or post-translational modification of Als3 was aberrant in the *ssa1*Δ/Δ mutant, we determined that deletion of *SSA1* had no effect on the amount and surface distribution of Als3 on *C. albicans* hyphae. These results suggest that proper Als3 trafficking can occur in the absence of Ssa1. Furthermore, the localization of Ssa1 on the fungal cell surface and the finding that beads coated with rSsa1 were avidly endocytosed suggests the more likely scenario that Ssa1 and Als3 function cooperatively as invasins.

After *C. albicans* invades host cells, it damages them. Host cell damage requires at least some extent of fungal invasion because blocking invasion by treatment with cytochalasin D or infection with non-invasive mutants of *C. albicans* reduces the extent of host cell damage [27], [31], [32], [50]. Thus, the defective host cell invasion of the *ssa1*Δ/Δ mutant likely contributed to its reduced capacity to damage these cells. Although the exact mechanism by which *C. albicans* causes host cell damage is incompletely understood, it is probable that phospholipases and aspartyl proteases secreted by the organism participate in this process [35], [40]–[46]. Some heat shock proteins can assist with protein secretion [47], [48]. However, we found no evidence of impaired phospholipase or protease secretion in the *ssa1*Δ/Δ mutant. While these results indicate that bulk protein secretion is intact in the *ssa1*Δ/Δ mutant, we cannot completely rule out the possibility that there was reduced secretion or activity of a single phospholipase or protease isozyme in this strain.

During both hematogenously disseminated and oropharyngeal infection, *C. albicans* is exposed to significant stress induced by the host. These stresses include exposure to reactive oxygen intermediates and antimicrobial peptides. Furthermore, in the oral cavity, *C. albicans* is likely exposed to toxic secondary metabolites produced by the resident oral flora. In some microbial pathogens, such as *Leishmania spp.* and *Francisella tularensis*, members of the Hsp70 and Hsp100 family of heat shock proteins are required for them to tolerate host-induced stress and cause persistent infection in mice [10]–[12]. Here, we found that Ssa1 was dispensable for the resistance of *C. albicans* to oxidant, cell wall, and cell membrane stress. We also determined that *ssa1*Δ/Δ mutant cells did not have increased susceptibility to killing by neutrophil-like HL-60 cells. Thus, the reduced virulence of the *ssa1*Δ/Δ mutant was unlikely due to impaired ability to withstand host-induced stress.

Previously we have found that Ssa1 acts as receptor for some antimicrobial proteins such as histatin 5, and that the *ssa1*Δ/Δ mutant is more resistant to some defensins [17]. Thus, one might have predicted that the *ssa1*Δ/Δ mutant would be resistant to neutrophil killing and therefore hypervirulent. However, the *ssa1*Δ/Δ mutant is not resistant to some toxic products of neutrophils, such as human neutrophil defensin 1 [17], reactive oxygen intermediates, and nitric oxide. Therefore, it is probable that the wild-type susceptibility of the *ssa1*Δ/Δ mutant to these stressors was the reason why it was neither resistant to killing by the neutrophil-like HL-60 cells in vitro nor hypervirulent in mice.

Although induction of host cell endocytosis is an important virulence function of Ssa1, it is possible that this protein may also contribute to *C. albicans* pathogenicity in other ways. For example, in *S. cerevisiae* Ssa1 regulates the transcription of the multidrug resistant gene, *PDR3* [53]. Furthermore, in *Cryptococcus neoformans*, Ssa1 functions as a transcriptional co-activator of laccase, and is required for normal melanin synthesis and virulence [54]. Thus, Ssa1 may either directly or indirectly govern the transcription of genes that also contribute to *C. albicans* pathogenicity. This possibility is currently being investigated.

## Materials and Methods

### Strains and growth conditions

The *C. albicans* strains used in these studies and their relevant genotypes are listed in Table 1. All strains were maintained on yeast extract/peptone/dextrose (YPD; Qbiogene) agar plates and re-cultured at least monthly from −80°C stock. For use in the experiments, yeast-phase cells of the various strains were grown YPD broth overnight in a rotary shaker at 30°C.

**Table 1:** Strains of *C. albicans* used in this study.

Strain	Genotype					Reference
SC5314	Wild-type					[55]
*ssa1*Δ/Δ	*ura3Δ::λimm434* *ura3Δ::λimm434*	*ssa1::FRT* *ssa1::FRT*	*ssa2::FRT::URA3::SSA2* *SSA2*			This paper
*ssa1Δ/Δ-URA3*	*ura3Δ::λimm434* *ura3Δ::λimm434*	*ssa1::FRT* *ssa1::FRT*	*ssa2::FRT* *SSA2*	*rps10::URA3* *RPS10*		This paper
*ssa1Δ*/*SSA1*	*ura3Δ::λimm434* *ura3Δ::λimm434*	*ssa1::FRT* *ssa1::FRT*	*ssa2::FRT* *SSA2*	*rps10::URA3::SSA1* *RPS10*		[16]
*ssa2Δ*/*Δ*	*ura3Δ::λimm434* *ura3Δ::λimm434*	*ssa1::FRT::URA3::SSA1* *SSA1*	*ssa2::FRT* *ssa1::FRT*			This paper
*Ssa2Δ*/*SSA2*	*ura3Δ::λimm434* *ura3Δ::λimm434*	*ssa2::FRT* *SSA1*	*ssa2::FRT* *ssa2::FRT*	*rps10::URA3::SSA2* *RPS10*		[16]
CAYF178U	*ura3Δ::λimm434::URA3-IRO1* *ura3Δ::λimm434*	*als3::ARG4* *als3::HIS1*	*arg4::hisG* *arg4::hisG*	*his1::hisG* *his1::hisG*		[68]
CAQTP178U	*ura3Δ::λimm434::URA3-IRO1* *ura3Δ::λimm434*	*als3::ARG4::ALS3* *als3::HIS1*	*arg4::hisG* *arg4::hisG*	*his1::hisG* *his1::hisG*		[68]
*ssa1Δ*/*Δ*-*als3Δ*/*Δ*	*ura3Δ::λimm434* *ura3Δ::λimm434*	*ssa1::FRT* *ssa1::FRT*	*ssa2::FRT* *SSA2*	*als3* *als3::NAT1*	*rps10::URA3* *RPS10*	This paper
*SSA1-GFP*	*ura3Δ::λimm434* *ura3Δ::λimm434*	*ssa1::SSA1-GFP::URA3* *SSA1*	*SSA2* *SSA2*			This paper

The initial *ssa1Δ/Δ*, *ssa2Δ/Δ*, *ssa1Δ/Δ::SSA1*, and *ssa2Δ/Δ::SSA2* strains were constructed previously [16]. To verify that the phenotype of the *ssa1*Δ/Δ mutant was not the result of *URA3* being integrated at the *SSA2* locus, a Ura- strain was selected by growth on 5-fluoroorotic acid [55]. This strain was transformed by the lithium acetate procedure with the CIp10 vector that had been linearized with NcoI [56]. The resulting strain, *ssa1*Δ/Δ-URA3 contained *URA3* at the neutral *RPS10* locus [21].

To delete the entire protein coding region of *ALS3* in the *ssa1*Δ/Δ mutant, the PCR-product-directed gene deletion approach was used [57]. Briefly, deletion cassettes containing *ALS3* flanking regions and the *URA3* or *NAT1* selection markers were amplified by PCR with primers GATATTTTGAATATGGAAATAAATCGTGCATAAGAAAGTTTTGCTATGCACGTTCATACTTCCAAAAATTGTAATACGACTCACTATAGGGC and AAACTATAGAAACAAACTAATCAAATTAACAACACACCAAATTGGAGGTAATTAATCATACCGAAAATAGCTATGACCATGATTACGCCA, using pGEM-URA3 [57] and pJK795 [58] as templates, respectively. These PCR products were then used to successively transform the Ura- ssa1*Δ*/*Δ* strain [57]. The resulting *ssa1*Δ/Δ *als3*Δ/Δ mutant was plated on 5-fluoroorotic acid to select for a Ura- strain. This strain was transformed with NcoI-linearized CIp10 [56] to re-integrated *URA3* at *RPS10* locus.

Strains containing C-terminal GFP fusions were generated in strain CAF4-2 (wt) by substitution of one *SSA1* allele with an *SSA1-GFP* tagged allele as described previously [16]. Plasmid pGFP-URA3 (PMG1602) was kindly provided by Dr. C. A. Gale, and was used as a template to PCR amplify the transformation cassettes using primers containing sequences flanking the *SSA1* stop codon (67 bp) and homologous sequences from the vector (forward primer: 9 bp glycine linker and 21bp vector sequence; reverse primer: 23 bp vector sequence). PCR products were used to transform parental strains using frozen EZ yeast transformation II kit (Zymo Research) following the manufacturer's protocol and selected on URA3^-^ YNB agar plates. To identify transformants carrying cassettes correctly integrated into the target gene sequence, genomic DNA was prepared and used as the template for PCR reactions using one primer for annealing within the transformation module and a second primer annealing with the target gene locus outside the altered region. Cells were examined using confocal microscopy and Western blotting with anti-GFP to confirm expression of Ssa1-GFP fusion protein.

### Mouse models of disseminated and oropharyngeal candidiasis

The virulence of the various strains was tested in the mouse model of hematogenously disseminated candidiasis as described previously [26]. Briefly, 10 male BALB/c mice (20 g body weight; National Cancer Institute, Bethesda, MD) were infected via the lateral tail vein with 5×10^5^ yeast-phase cells of each strain of *C. albicans* in 500 µl of PBS. All inocula were confirmed by quantitative culture. The mice were monitored at least three times daily, and moribund mice were euthanized. To determine the organ fungal burden, 5 to 7 mice were inoculated with each strain as in the survival experiments. After 4 h, 8 h, 1 day, 2 days, 4 days, 7 days, and 14 days of infection, the brain, liver, and one kidney from each mouse were harvested, weighed, homogenized, and quantitatively cultured. The remaining kidneys from the 1 day time point were fixed in zinc-buffered formalin followed by 70% ethanol and then embedded in paraffin. Thin sections were cut and stained with periodic acid-Schiff.

The different strains of *C. albicans* were also tested for virulence in our previously described mouse model of oropharyngeal candidiasis [25], [27], [59]. Briefly, male BALB/c mice were immunosuppressed with cortisone acetate (225 mg/kg; Sigma-Aldrich) administered subcutaneously on days -1, 1, and 3 relative to infection. For oral inoculation, each mouse was anaesthetized by intraperitoneal injection with ketamine and xylazine (both from Phoenix Pharmaceuticals), after which a calcium alginate swab (Type 4 Calgiswab; Puritan Medical Products Company LLC) saturated with 10^6^ yeast/ml was placed sublingually for 75 min. The mice were subsequently provided food and water ad libitum and then sacrificed after 1, 2 and 5 days of infection. The tongue and adjacent hypoglossal tissue were excised and cut in half. One half was weighed and homogenized for quantitative culture and the other half was processed for histopathological analysis as described above.

### Endothelial cells and oral epithelial cells

Endothelial cells were isolated from human umbilical cord veins by the method of Jaff et al. [60] and cultured as described [32] in M-199 medium supplemented with 10% fetal bovine serum and 10% defined bovine calf serum (Gemini Bio-Products), and containing 2 mM L-glutamine with penicillin and streptomycin (Irvine Scientific). Endothelial cells were used at the third passage. The FaDu oral epithelial cell line (American Type Culture Collection) was cultured in Eagle's minimum essential medium with Earle's balanced salt solution (Irvine Scientific) supplemented with 10% fetal bovine serum, 1 mM pyruvic acid, 2 mM l-glutamine, and 0.1 mM nonessential amino acids, with penicillin and streptomycin [25], [27]. Both cell types were maintained in a humidified incubator in 5% CO_2_ at 37°C.

### Host cell damage assay

The extent of endothelial and epithelial cell damage caused by the different strains of *C. albicans* was measured using our previously described ^51^Cr release assay [25]–[27], [31], [32]. Briefly, endothelial cells or FaDu oral epithelial cells were grown to 95% confluency in 96 well tissue culture plates with detachable wells (Corning) and loaded with 5 µCi/ml Na_2_^51^CrO_4_ (MP Biomedicals) overnight. After removing the unincorporated ^51^Cr by rinsing, the cells were then infected with yeast of the various *C. albicans* strains suspended in RPMI 1640 medium (Irvine Scientific). The inoculum was 4×10^4^ organisms per well of endothelial cells and 1×10^5^ organisms per well of oral epithelial cells. The infected host cells were incubated for 3 h, after which the amount of ^51^Cr released into the medium and retained by the cells was determined by γ-counting. Wells containing host cells, but no organism, were processed in parallel to determine the spontaneous release of ^51^Cr. After correcting for well-to-well differences in the incorporation of ^51^Cr, the percent specific release of ^51^Cr was calculated using the following formula: (experimental release - spontaneous release)/(total incorporation - spontaneous release). Experimental release was the amount of ^51^Cr released into the medium by cells infected with *C. albicans*. Spontaneous release was the amount of ^51^Cr released into the medium by uninfected host cells. Total incorporation was the sum of the amount of ^51^Cr released into the medium and remaining in the host cells. Each assay was performed in triplicate on three separate occasions.

### Three-dimensional model of the human oral mucosa

The capacity of the various *C. albicans* strains to invade a three dimensional model of the oral mucosa in vitro was determined by our previously described method [34], [61]. OKF6/TERT-2 oral epithelial cells were grown on top of a feeder layer of collagen embedded NIH 3T3 cells in 30 mm diameter cell culture inserts (Millipore). They were infected via their apical surface by adding 10^5^ *C. albicans* cells in 100 µl of airlift medium (DMEM with 4.5 g/l glucose [Fisher Scientific] and Ham's F-12 medium [Invitrogen] mixed 3∶1, and supplemented with 5 µg/ml insulin, 0.4 µg/ml hydrocortisone, 2×10^11^ M 3,3′,5-triiodo-L-thyronine, 1.8×10^−4^ M adenine, 5 µg/ml transferrin, 10^−10^ M cholera toxin, 2 mM L-glutamine; 5% FBS, and penicillin–streptomycin). For histopathology, the cultures were fixed after 2 days with 10% formaldehyde in PBS and embedded in paraffin. Thin sections were stained with periodic acid-Schiff and then evaluated by light microscopy. The extent of epithelial cell damage was quantified after 2 days of infection by measuring the accumulation of LDH in the medium using the CytoTox-96 assay (Promega).

### Adherence and endocytosis assay

The capacity of the various *C. albicans* strains to adhere to and be endocytosed by endothelial and FaDu epithelial cells was quantified using our previously described differential fluorescence assay [25], [27], [32], [36]. The host cells were grown to 95% confluency on fibronectin-coated glass coverslips in a 24-well tissue culture plate. Each coverslip was infected with 10^5^ organisms in RPMI 1640 medium. After a 90 or 150 min incubation, the medium was aspirated, non-adherent organisms were removed by rinsing the coverslips with Hank's balanced salt solution (HBSS; Irvine Scientific), and cells were fixed with 3% paraformaldehyde. The adherent and non-internalized portions of the organisms were stained with rabbit anti-*C. albicans* antiserum (Biodesign International) conjugated with Alexa 594 (Molecular Probes). Next, the host cells were permeabilized with 1% Triton X-100 for 15 min, and then all of the *C. albicans* cells were stained with rabbit anti-*C. albicans* antiserum conjugated with Alexa 488 (Molecular Probes). The coverslips were mounted inverted on a microscope slide and organisms were viewed under epifluorescence. The number of endocytosed organisms was determined by subtracting the number of non-endocytosed organisms (which fluoresced red) from the number of cell-associated organisms (endocytosed plus non-endocytosed organisms, which fluoresced green). Organisms that were partially internalized were considered to be endocytosed. At least 100 organisms were counted per coverslip, and each experiment was repeated in triplicate, at least three times. Results were expressed as the number of endocytosed and cell-associated organisms per high powered field.

### *C. albicans* binding to endothelial cell N-cadherin and epithelial cell E-cadherin

The capacity of each strain to bind to N-cadherin and E-cadherin was determined using our affinity purification procedure as outlined previously [36], [37]. Germ tubes were prepared by incubating yeast-phase cells for 90 min at 37°C in 150 mm diameter Petri dishes containing RPMI 1640 medium buffered to pH 7.5 with 150 mM HEPES. The resulting germ tubes were removed by scraping and rinsed once with PBS containing calcium and magnesium. The germ tubes (2×10^8^ cells) were incubated for 1 h on ice with 250 µg of cell membrane proteins of endothelial cells or FaDu epithelial cells in PBS with calcium and magnesium containing 1.5% octyl-glucopyranoside and protease inhibitors. The unbound proteins were removed by rinsing in the same buffer, after which the proteins that remained bound to the germ tube were eluted with 6 M urea. The eluted proteins were separated by SDS-PAGE. N-cadherin and E-cadherin were detected by immunoblotting with anti-N-cadherin murine monoclonal antibody (clone 32, Transduction Laboratories), and an anti-E-cadherin murine monoclonal antibody (clone HECD 1), respectively.

### Host cell interactions of latex beads coated with rSsa1, rSsa2, or BSA

Recombinant Ssa1 (rSsa1) and rSsa2 were produced in *S. cerevisiae* as outlined previously [16]. Latex beads were coated with these proteins or biotinylated BSA as described [37], [62]. Briefly, 5×10^7^ fluorescent, yellow-green, amine-modified, 2.0 µm diameter polystyrene latex beads (Sigma-Aldrich) were washed with PBS followed by coupling buffer (0.2 M Na_2_HCO_3_ [pH 8.5], 0.5 M NaCl). The beads were then incubated with rSsa1, rSsa2 (0.5 mg/ml) or coupling buffer at 37°C for 30 min. The beads that had been coated with Ssa proteins were incubated with 1% rabbit serum, while the control beads were incubated with biotinylated BSA (1 mg/ml). All beads were sonicated briefly, blocked with unlabeled BSA and rinsed PBS-BSA. They were suspended in PBS containing 2 mg BSA per ml. Binding of rSsa1 or rSsa2 to the beads was verified by indirect immunofluorescence with an anti-Xpress monoclonal antibody (Invitrogen) directed against the Xpress leader sequence of these recombinant proteins. Binding of biotinylated BSA to the beads was confirmed using Alexa 568 conjugated streptavidin (Invitrogen). The interactions of these beads with endothelial and FaDu epithelial cells were determined by the differential fluorescent assay described above.

### Real-time PCR detection of *SSA1* and *SSA2* transcript levels

The relative transcript levels of *SSA1* and *SSA2* in hyphae of the various strains of *C. albicans* were determined by our previously described method [63]. Yeast cells were suspended in RPMI 1640 medium and added to FaDu epithelial cells at a final concentration of 5×10^5^ cells/cm^2^. After a 90 min incubation, the non-adherent organisms were removed by rinsing with ice-cold distilled water. Next, ice-cold DEPC-treated water was added and the *C. albicans* and epithelial cells were removed with a cell scraper. The mixture was vortexed for 30 sec to lyse the epithelial cells and then the organisms were collected by a brief centrifugation at 4°C. These organisms were suspended in TES buffer (10 mM Tris, 10 mM EDTA, 0.5% SDS), and then snap frozen in liquid nitrogen. The total time from rinsing the organisms to freezing them in liquid nitrogen was less than 5 min. At a later time, the cells were thawed on ice and the fungal RNA was extracted by the hot phenol method [64]. For real-time PCR, the *C. albicans* RNA was treated with DNase I (Ambion), after which cDNA was synthesized using MMLV reverse transcriptase (Ambion). Quantitative real-time PCR was carried out using the SYBR green PCR kit (Applied Biosystems) and an ABI 7000 Real-Time PCR System (Applied Biosystems) following the manufacturer's protocol. The results were analyzed by the 2^-ΔΔCT^ method [65] using *ACT1* as the endogenous control. The *SSA1* and *SSA2* transcript levels were determined in three biological replicates, each tested in duplicate.

### Assessment of surface expression of Als3 and Ssa1

Flow cytometry was used to quantify the amount of Als3 on the surface of the various strains using a slight modification of our previously described method [37]. Briefly, 3×10 10^6^ yeast-phase cells in 15 ml RPMI 1640 medium buffered to pH 7.5 with 10 mM HEPES were added to 100 mm diameter Petri dishes and incubated in 5% CO_2_ at 37°C. After 90 min, the resulting germ tubes were scraped from the dishes with a cell scraper, fixed with 3% paraformaldehyde, blocked with 1% goat serum, and then stained with a rabbit polyclonal anti-Als3 antiserum [37] followed by an Alexa 488 conjugated goat anti-rabbit antibody (Molecular Probes). An *als3*Δ/Δ mutant was included in these experiments as a negative control. The amount of Als3 surface staining on 10^4^ germ tubes per strain was analyzed by flow cytometry.

To analyze the surface distribution of Als3 on the various strains, 10^5^ yeast-phase cells in 1 ml RPMI 1640 medium buffered to pH 7.5 with 10 mM HEPES were added to 12 mm diameter glass cover slips in a 24-well tissue culture plate. After a 90 min incubation in 5% CO_2_ at 37°C, the resulting germ tubes were fixed with 3% paraformaldehyde, and blocked with 1% goat serum, and then stained with the rabbit polyclonal anti-Als3 antiserum followed by an Alexa 488 conjugated goat anti-rabbit antibody. Next, the germ tubes were counter stained with the Alexa 594 conjugated anti-*Candida* antibody to label the cell surface. The coverslips were mounted inverted on microscope slides and imaged by confocal microscopy. Stacked images were acquired along the z-axis and then combined for the final images.

The surface expression of Ssa1 on *C. albicans* expressing Ssa1-GFP was determined by a similar process, except that FaDu oral epithelial cells were grown on the coverslips prior to adding the organisms and the anti-Als3 antiserum was omitted.

### Screening for extracellular phospholipase and protease activities

The *C. albicans* strains were screened for phospholipase activity by the method of Samaranayake et al. [49]. Briefly, egg yolk agar consisting of Sabouraud's dextrose agar, NaCl, CaCl_2_ and egg yolk emulsion was prepared. A 10 µl of suspension of 10^7^ yeast-phase cells per ml in 10 mM sodium phosphate buffer was plated on the agar and incubated at room temperature for 5 days. The phospholipase activity of each strain was determined by measuring the width of zone of precipitation around the colony.

The total extracellular protease activity of the various strains was assessed using BSA agar by the method of Ruchel et al. [66]. BSA agar (0.2% BSA, 1.17% yeast carbon base, and 0.01% yeast extract, pH 5) was spot inoculated with the various *C. albicans* strains and then incubated at 37°C. After 5 days the agar was stained with 0.5% amido black and the width of the zone of clearance around each colony was measured.

### Susceptibility to stressors

The susceptibility of the various *C. albicans* strains to stressors were tested by spotting dilutions of 10^4^ to 10^1^ yeast-phase cells in a total volume of 7 µl onto YPD agar plates containing menadione (12.5 and 25 µM); H_2_O_2_ (1, 2 and 4 mM), Calcafluor white (25 and 50 µM), NaCl (1 and 2 M) and SDS (0.002%). The plates were incubated at 30°C for 24 hours and then imaged.

The susceptibility of the different strains to damage by a neutrophil-like cell line was determined by the 2,3-bis (2-methoxy-4-nitro-5-sulfophenyl)-5-[(phenylamino) carbonyl]-2H-tetrazolium hydroxide (XTT) assay, as previously described [67]. Briefly, HL-60 cells were cultured in RPMI 1640 medium containing 10% fetal bovine serum and 25 mM HEPES. They were induced to differentiate into neutrophil-like cells by exposure to 1.25% dimethyl sulfoxide for 7 days. For the damage assay, *C. albicans* yeast suspended in Dulbecco's Modified Eagle Medium containing 10% fetal bovine serum were added to 96-well plates at concentrations ranging from 10^5^ to 2×10^4^ cells/well. There was a linear relationship between viable cell number and colorimetric signal (XTT activity) in this concentration range with all three strains (not shown). HL-60 cells were added to the *C. albicans* cells at effector to target cell ratios (E:T) ranging from 5∶1 to 1∶2. After incubation at 37°C in 5% CO_2_ for 3 hours, the medium was aspirated and the HL-60 cells were lysed with sterile H_2_O. To each well was added 100 µl of a mixture of XTT and coenzyme Q0 (0.25 mg/ml XTT and 40 µg/ml coenzyme Q0), after which the plate was incubated at 37°C in 5% CO_2_ for 2 h. Supernatants were transferred into new plates, and optical densities (OD) were measured by an Opsys Microplate Reader (Thermo Labsystems) at 450–490 nm, with a 630 nm reference filter. Antifungal activity was calculated according to the following formula: %fungal damage  =  (1−x/n)*100, where x is the OD450 of experimental wells (*C. albicans* with effectors) and n is the OD450 of control wells (*C. albicans* only). Each experiment was performed in triplicate and repeated 3 times.

### Statistics

The results of the survival experiments were analyzed with the Log-Rank Test, and the organ fungal burden data were analyzed using the Log Rank Test. The results of the in vitro experiments were analyzed using the Student's T test. *P* values ≤0.05 were considered to be significant.

### Ethics statement

All experiments were approved by the Los Angeles Biomedical Research Institute Animal Care and Use Committee and as outlined in the Guide for the Care and Use of Laboratory Animals of National Institutes of Health. The collection of umbilical cords for the harvesting of endothelial cells used in these studies was approved by the Institutional Review Board of the Los Angeles Biomedical Research Institute at Harbor-UCLA Medical Center.

## Supporting Information

Figure S1 The chromosomal location of *URA3* does not influence the virulence or host cell interactions of the *ssa1*Δ/Δ mutant. (A). Survival of mice infected with 5×10^5^ yeast cells of the indicated strains of *C. albicans*. Each strain was used to inoculate 10 mice. (B) Endothelial cells and FaDu oral epithelial cells were incubated with the indicated strains for 90 min, after which the number of endocytosed and cell-associated organisms were determined. Results are the mean ± SD of 3 experiments, each performed in triplicate.
(0.45 MB TIF)

Figure S2 Localization of Ssa1 on *C. albicans* yeast. *C. albicans* yeast expressing an Ssa1-GFP fusion protein were stained with an Alexa 594-conjugated anti-*C. albicans* polyclonal antibody to label the cell surface and then imaged by confocal microscopy. (A–C) Images of Ssa1-GFP (A) and the fluorescent-labeled anti-*C. albicans* antibody (B). The merged image is shown in (C). (D) Graphs of fluorescent intensity at different cross sections of the yeast in panel (C). The green lines indicate the fluorescent intensity of the Ssa1-GFP and the red lines indicate the fluorescent intensity of the fluorescent-labeled anti-*C. albicans* antibody.
(1.38 MB TIF)
