# Supplementary figures and images for: Host Cell Invasion and Virulence Mediated by Candida albicans Ssa1
Source: PLoS Pathog. 2010 Nov 11;6(11):e1001181. doi: 10.1371/journal.ppat.1001181 (PMC2978716; doi:10.1371/journal.ppat.1001181)

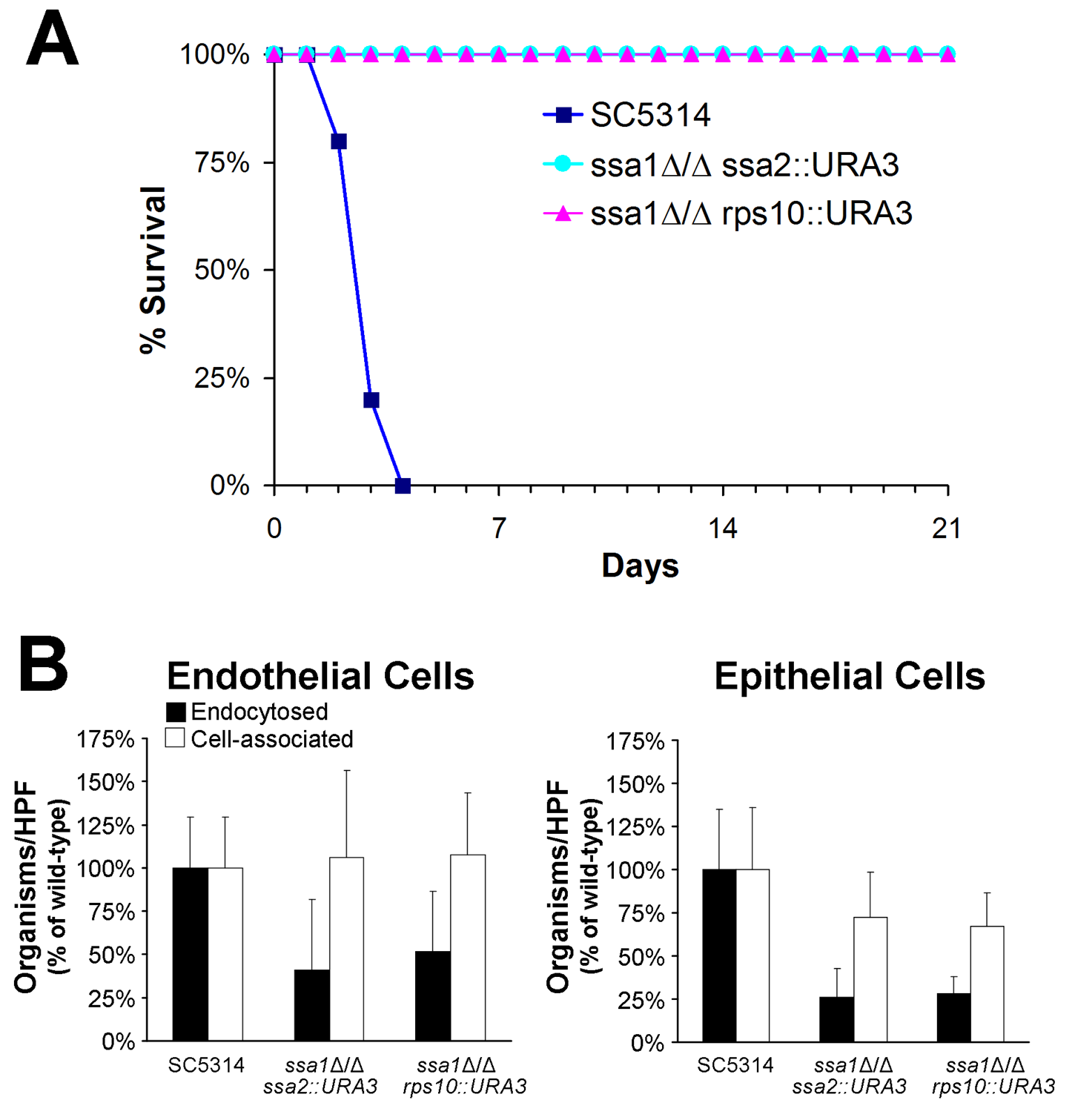

Supplement: Figure S1 — The chromosomal location of URA3 does not influence the virulence or host cell interactions of the ssa1Δ/Δ mutant. (A). Survival of mice infected with 5×10^5^ yeast cells of the indicated strains of C. albicans. Each strain was used to inoculate 10 mice. (B) Endothelial cells and FaDu oral epithelial cells were incubated with the indicated strains for 90 min, after which the number of endocytosed and cell-associated organisms were determined. Results are the mean ± SD of 3 experiments, each performed in triplicate. (0.45 MB TIF) [file ppat.1001181.s001.tif]

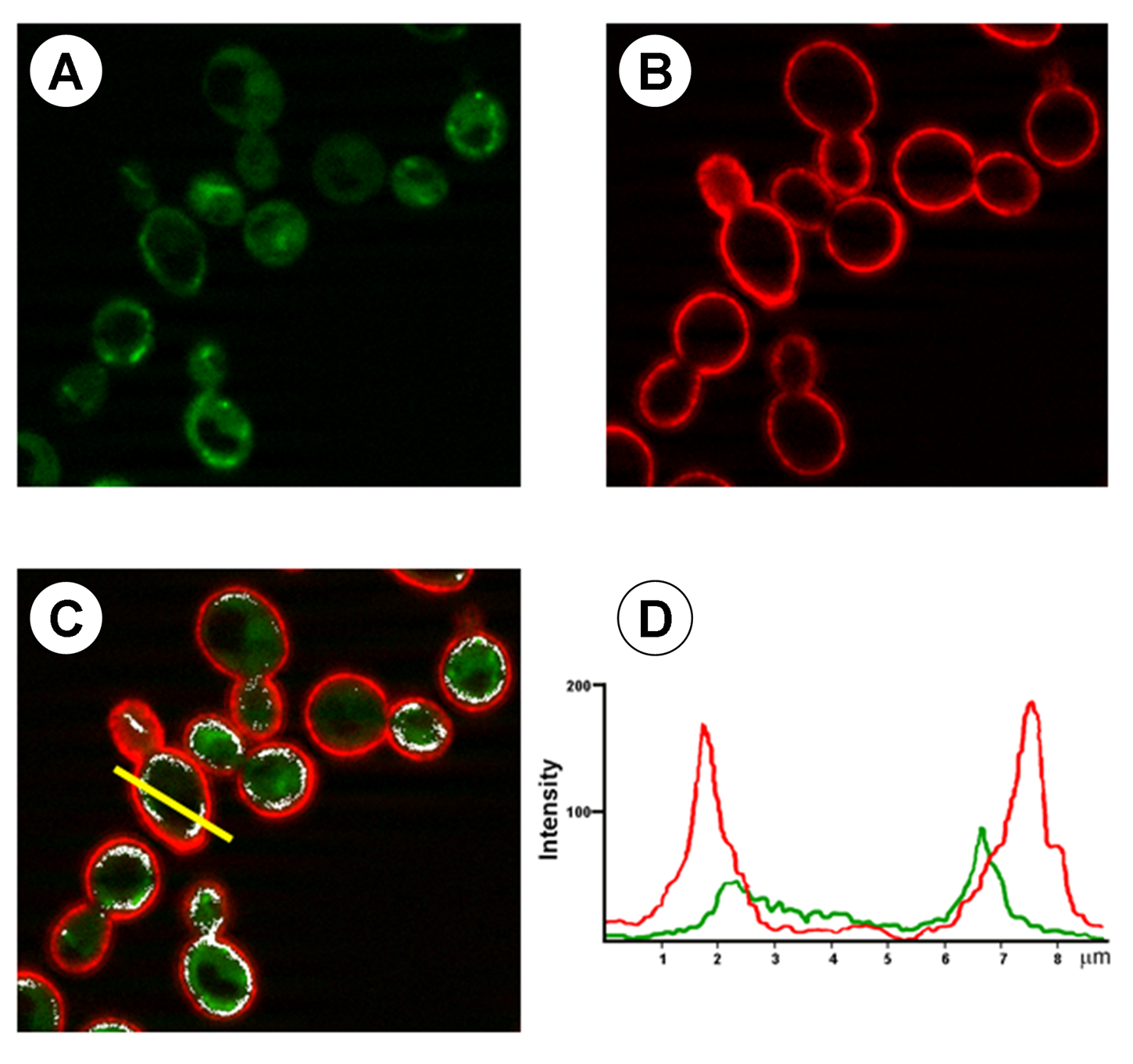

Supplement: Figure S2 — Localization of Ssa1 on C. albicans yeast. C. albicans yeast expressing an Ssa1-GFP fusion protein were stained with an Alexa 594-conjugated anti-C. albicans polyclonal antibody to label the cell surface and then imaged by confocal microscopy. (A–C) Images of Ssa1-GFP (A) and the fluorescent-labeled anti-C. albicans antibody (B). The merged image is shown in (C). (D) Graphs of fluorescent intensity at different cross sections of the yeast in panel (C). The green lines indicate the fluorescent intensity of the Ssa1-GFP and the red lines indicate the fluorescent intensity of the fluorescent-labeled anti-C. albicans antibody. (1.38 MB TIF) [file ppat.1001181.s002.tif]
